# Efficacy and safety of limb distalization after Roux-en-Y gastric bypass: a retrospective cohort study

**DOI:** 10.3389/fsurg.2026.1860731

**Published:** 2026-08-04

**Authors:** Egon Rodrigues, Mónica Bandeira, Rui Ferreira de Almeida, Mário Nora, Mariana P. Monteiro, Sofia S. Pereira, Marta Guimarães

**Affiliations:** 1 Department of General Surgery, Local Health Unit Entre o Douro e Vouga, Santa Maria da Feira, Portugal; 2 UMIB - Unit for Multidisciplinary Research in Biomedicine, ICBAS - School of Medicine and Biomedical Sciences, University of Porto, Porto, Portugal; 3 ITR - Laboratory for Integrative and Translational Research, Porto, Portugal

**Keywords:** limb distalization, nutritional deficiency, obesity, Roux-en-Y gastric bypass, weight loss

## Abstract

**Introduction:**

Limb distalization (LD) is an option for highly selected patients with suboptimal clinical response after Roux-en-Y gastric bypass (RYGB). This study assessed the effectiveness and safety of LD in a cohort of post-RYGB patients with suboptimal results, focusing on weight loss, obesity-related diseases remission, and nutritional outcomes.

**Methods:**

Retrospective observational study of patients who underwent LD between 2011 and 2023 at our institution. Only patients with recurrent weight gain after RYGB were selected. Outcomes assessed included the percentage of total weight loss (%TWL), percentage of excess weight loss (%EWL), remission of obesity-related diseases, and morbidity. Nutritional deficiencies were also evaluated.

**Results:**

A total of 22 patients underwent LD. The interval between RYGB and LD was 116.8 months. The average BMI pre-LD was 39.0 kg/m^2^. At 12 months, mean %EWL and %TWL were 58.2% and 19.6%, respectively. At 60 months or more, %EWL was 49.9%, and %TWL was 19%. Remission of type 2 diabetes mellitus, dyslipidemia, and arterial hypertension occurred in 75%, 60%, and 40% of patients, respectively. The rate of early complications was 0%, while the rate of late complications was 27%. Protein malnutrition occurred in 9.1% of patients, with no cases of reversal surgery.

**Conclusion:**

LD was linked to substantial and sustained weight loss and significant remission of obesity-related metabolic diseases. Nutritional deficiencies remain a concern, particularly in patients with shorter total alimentary limb lengths, emphasizing the importance of individualized limb length and close follow-up.

## 1 Introduction

The 8th International Federation for the Surgery of Obesity and Metabolic Disorders Global Registry Report states that around 142.000 Roux-en-Y gastric bypasses (RYGB) were performed in 2023 (1). RYGB is a highly effective long-term procedure for treating obesity and related diseases. However, it is a recognized problem that some patients experience an unsatisfactory response to surgery (2, 3). The reported prevalence of unsatisfactory response varies widely across studies, mainly due to inconsistent definitions in the literature (3–5). Approximately 30% of the patients who underwent RYGB as primary metabolic and bariatric surgery (MBS) at our institution had a suboptimal clinical response, defined as losing less than 20% of the total weight 10 or more years after surgery (6).

The suboptimal weight loss can be attributed to a multitude of factors that can interact in very complex ways (2, 5). An appropriate multidisciplinary approach is essential. A secondary MBS may be considered for highly selected patients.

According to the recent Delphi consensus, these surgeries should be categorized as conversion if the procedure converts to a new type of MBS, such as duodenal switch ± biliopancreatic diversion (DS ± BPD) and single anastomosis duodeno-ileal bypass with sleeve (SADI-S), reversal when it restores normal anatomy, and revision if it modifies or enhances the effects of a previous operation, namely limb distalization (LD) (7). Procedures after RYGB were traditionally divided as restrictive or hypoabsorptive (8), with DS ± BPD, SADI-S and LD falling into the latter category. These procedures are associated with greater and sustained weight loss, but they also pose a higher risk of complications (9). The conversion to DS ± BPD or SADI-S entails a longer surgical time (10, 11) and a higher likelihood of complications, as it requires the restoration of anatomy to its pre-RYGB state before performing the required anastomoses (12). Despite the fact of LD being the most common hypoabsorptive revision for primary RYGB (8), studies reporting its effectiveness typically encompass a small number of patients, since it is still rarely performed. Therefore, our aim was assess the efficacy and safety of LD in patients with recurrent weight gain after RYGB and contribute with original data to fill this knowledge gap.

## 2 Materials and methods

### 2.1 Design and subjects

This study was a retrospective observational cohort study of patients who underwent LD after RYGB at our institution between March 2011 and June 2023. The study was designed and reported conforms to the Strengthening the Reporting of Observational Studies in Epidemiology (i.e., STROBE) statement (13). Patients were considered eligible for LD whenever they had recurrent weight gain, if conservative interventions had been unsuccessful, and after undergoing a multidisciplinary evaluation. Patients with anatomical alterations such as pouch dilation, gastrojejunal anastomosis dilation, or gastrogastric fistula requiring surgical intervention other than LD were excluded. All included patients had a follow-up period of more than 6 months after LD. Suboptimal clinical response was defined as a percentage of total weight loss (%TWL) under 20% at nadir after RYGB, while recurrent weight gain was defined as gain of more than 30% of the initial surgical weight loss (7).

### 2.2 Preoperative evaluation

Endocrinologists, dieticians, psychologists, and bariatric surgeons evaluated all patients undergoing revisional surgery prior to the procedure. The preoperative study included an endoscopy and a gastrointestinal contrast study to rule out anatomical abnormalities.

### 2.3 Surgical technique

All surgeries were performed by the same team of experienced bariatric surgeons. The identification and measurement of limbs were performed using a graduated grasper with measurement of the entire small intestine. Type I LD (8) was conducted by detaching the alimentary limb (AL) from the jejunojejunostomy and reattaching it closer to the terminal ileum with reconfection of the Roux-en-Y anastomosis, increasing the length of the biliopancreatic limb (BPL) and keeping the length of the AL. The decision to use this surgical approach was grounded on previous evidence of the impact of BPL length in weight loss and obesity-related diseases improvement (14–16).

### 2.4 Postoperative follow-up

After surgery, patients were advised to follow a micronutrient supplementation plan consisting of a commercially available post-bariatric oral multivitamin formulation on a daily basis and oral cholecalciferol (506 µg) and intramuscular B complex vitamins (100 mg of thiamine hydrochloride, 100 mg of pyridoxine hydrochloride, and 1 mg of cyanocobalamin), monthly. Follow-up visits were scheduled one week after surgery and every 3 months during the first postoperative year, with alternating visits to the surgeon and the endocrinologist, and biannually thereafter. The routine assessment also included biannual appointments with a psychologist and a dietician. Full blood count and serum vitamin levels assessments were carried out at 3, 6, and 12 months during the first year after surgery and then biannually.

### 2.5 Outcomes

The primary outcome was weight loss, defined as the %TWL, and the percentage of excess weight loss (%EWL).

The secondary outcomes were remission of obesity-related diseases and the occurrence of any complication. The obesity-related diseases assessed included type 2 diabetes mellitus (T2D), arterial hypertension (AHT) and dyslipidemia. T2D was defined as having a HbA1c >6.5% or a use of antidiabetic medication. AHT was defined as systolic pressure >140 mmHg and/or a diastolic blood pressure >90 mmHg or a use of antihypertensive medication. Dyslipidemia was defined as an elevated cholesterol and/or triglyceride level or a use of any lipid lowering medication. Remission was defined as the absence of diagnostic criteria for each condition without pharmacological treatment (17). Vitamin and mineral deficiencies were defined as serum levels below the lower limit of normal according to our institutional laboratory reference values. Albumin deficiency was defined as serum albumin level <3.4 g/dL. Surgical complications were classified as early when they occurred within 90 days postoperatively or as late when they occurred beyond 90 days.

### 2.6 Statistics

Categorical variables were quantified as the number of cases and percentage (%) and were compared between the timepoints using the chi-squared test. Continuous variables were reported as mean ± standard deviation, or median with interquartile range, as applicable. The statistical analysis to assess differences over time was performed using linear mixed model with covariance type autoregression 1 and time as fixed effect and was reported as mean ± standard error of mean. Spearman's correlation coefficient was used to assess correlation between daily bowel movements and serum albumin levels. Statistical significance was defined by *p* < 0.05. SPSS version 29.0.0.0 was used for statistical analysis.

## 3 Results

A total of 22 patients with an average age of 46.9 ± 7.5 years underwent revisional LD surgery between March 2011 and June 2023. Twenty-one (95.5%) were female. All but one had been submitted to RYGB at our institution. At the time of index surgery, the mean age was 38 ± 8.7 years, the mean weight was 123.3 ± 13.3 kg, and the mean BMI was 45.9 ± 5.5 kg/m^2^. Three patients were submitted to a RYGB variant named metabolic gastric bypass, using a 200 cm long BPL. AL has a fixed length of 120 cm, in our technique.

After the initial surgery, weight decreased significantly (*p* < 0.001), reaching a minimum of 83.4 ± 14.9 kg, which represented a reduction in %TWL and %EWL of 32.1 ± 10.7% and 71.9 ± 21.5%, respectively. All patients achieved a %TWL > 20% at nadir. The nadir BMI was 31.0 ± 5.2 kg/m^2^ (*p* < 0.001).

The interval between surgeries was 116.8 ± 51.0 months (minimum 43, maximum 216). In addition to all the patients experienced recurrent weight gain–median weight regain was 53.6% (IQR: 46.6–62.8), 3 also had a resurgence of obesity-related diseases that had previously been in remission (namely, 2 cases of AHT and 1 case of T2D). At the time of LD, average %TWL and %EWL were 14.4 ± 2.5% and 31.4 ± 5.6%, respectively.

All LD surgeries were performed laparoscopically and had a median operative time of 76 min (IQR: 64.5–82). The final limb lengths varied and were adjusted for each patient, as described in Table 1. The length of in hospital stay was 2 days (IQR: 2–3).

**Table 1:** Intestinal limbs lengths after LD, cm.

Intestinal limb	Median	Interquartile range	Minimum	Maximum
AL	180	100–200	75	210
CL	160	100–200	100	300
TALL	300	285–315	220	500
BPL	Not systematically available			

AL, alimentary limb; BPL, biliopancreatic limb; CL, common limb; LD, limb distalization; TALL, total alimentary limb length.

The median follow-up was 43.5 months (IQR: 27–86), with no dropouts. Sixteen patients (72.7%) completed 36 months of follow-up, while nine (40.9%) reached 60 months or more.

**Figure 1:**
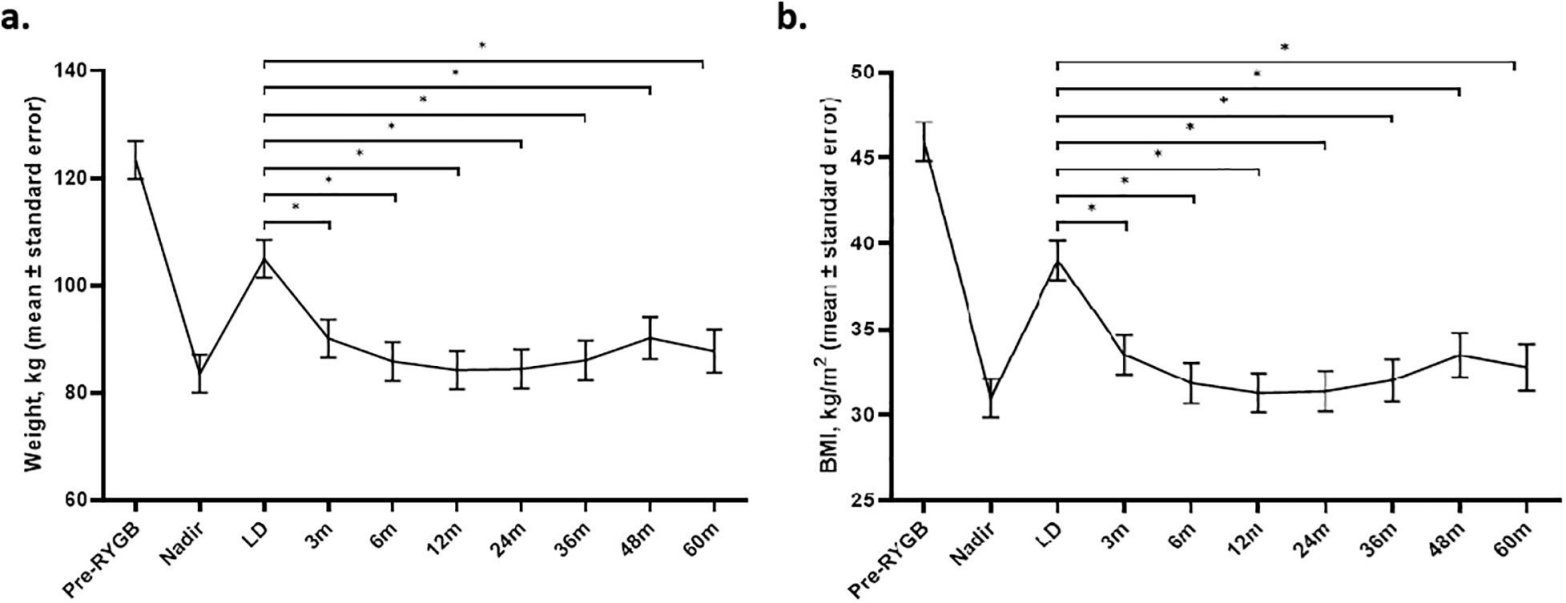
**(a)** Weight and **(b)** body mass index (BMI) prior to limb distalization (LD) and after at different follow-up times (3, 6, 12, 24, 36, 48 and 60 months or more). * *p* < 0.001.

**Figure 2:**
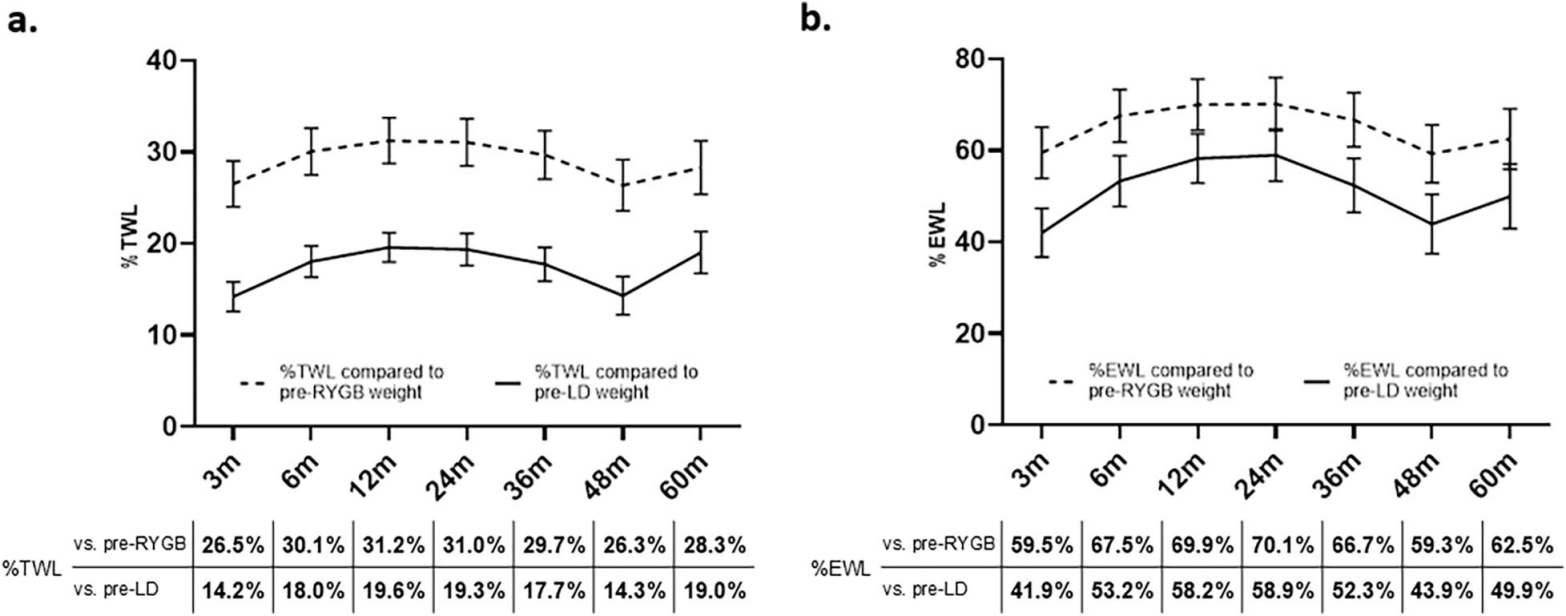
**(a)** % of total weight loss (%TWL) and **(b)** % of excess weight loss (%EWL) progression after limb distalization compared to pre-RYGB weight and pre-LD weight. No statistically significant differences were observed between timepoints.

The weight loss and BMI after surgery are shown in Figure 1. The LD resulted in a statistically significant reduction in the estimated marginal means from pre-LD (weight 104.9 ± 3.5 kg and BMI 39.0 ± 1.1 kg/m^2^) to 12 months (84.2 ± 3.5 kg and 31.3 ± 1.1 kg/m^2^, *p* < 0.001) and to 60 months or more (87.8 ± 4.0 kg and 32.8 ± 1.4 kg/m^2^, *p* < 0.001). The average %TWL and %EWL over time after surgery are shown in Figure 2. Both variables showed an increasing trend up to 24 months, after which they either plateaued or decreased. When compared to pre-LD weight the %TWL at 12, 24, and 60 or more months was 19.6%, 19.4%, and 19.0%, respectively, while the %EWL was 58.2%, 58.9%, and 49.9%. When compared to pre-RYGB weight, the %TWL at 12, 24, and 60 or more months was 31.2%, 31.0%, and 28.3%, and the %EWL was 69.9%, 70.1%, and 62.5%, respectively. All but one (maximum %EWL of 49.33%) exhibited a %EWL > 50% at some point during the follow-up after LD, and all had a %TWL > 20%, when these variables were calculated using the pre-RYGB weight as reference. When calculating %EWL and %TWL using the pre-LD weight as reference, it was found that 18.2% (*n* = 4) did not reach %EWL > 50% and 31.8% (*n* = 7) did not achieve %TWL > 20%, although all of them recorded a %TWL > 10%.

Regarding obesity-related diseases, after LD there was a remission rate of 75% for T2D, 60% for dyslipidemia, and 40% for AHT (Table 2).

**Table 2:** Obesity-related diseases evolution.

Obesity-related disease	Pre-RYGB	Pre-LD	Follow-up after LD	
			Remission	Recurrence
Arterial Hypertension	9	5	5 (100%^a^)	3 (60%^b^)
Dyslipidemia	9	10	7 (70%^a^)	1 (14%^b^)
Type 2 Diabetes Mellitus	4	4	4 (100%^a^)	1 (25%^b^)

LD, limb distalization; RYGB, Roux-en-Y gastric bypass.

a Value calculated in relation to the pre-LD number.

b Value calculated in relation to the number of patients with remission post-LD.

Concerning micronutrient levels shown in Table 3, the most frequent chronic deficiency was vitamin D. At some point during follow-up, five patients reported more than 4 bowel movements per day, and two of them developed a marked protein malnutrition (albumin <2.9 g/dL) at 6 months of follow-up. One patient required prolonged hospitalization with parenteral nutrition. However, the daily bowel movements showed a very weak and non-significant correlation (rs = −0.0073, *p* = 0.592) with serum albumin levels in our population. The rates of early and late complications excluding nutritional deficiencies previously mentioned, were 0% and 27.3%, respectively. Complication rates are shown in Table 3.

**Table 3:** Late complications including nutritional deficiencies.

Post-operative late complications										
			*N*				%			
Symptomatic gallstone disease			3^a^				13.6			
Dumping syndrome			2^a^				9.1			
Internal hernia (Brolin space)			1				4.5			
Anastomotic ulcer			1				4.5			

**Nutritional deficiencies**										
		RYGB	LD	3 M	6 M	12 M	24 M	36 M	48 M	60 M
	Total N	22	22	22	22	22	19	16	13	9
Anemia	Valid N	13	22	19	15	21	16	13	7	9
	Valid Percent	7.7%	22.7%	21.1%	40%	45.9%	56.3%	53.8%	42.9%	44.4%
Iron	Valid N	3	17	19	15	20	15	11	6	8
	Valid Percent	0%	29.4%	31.6%	20%	30%	9.1%	33.3%	33.3%	0%
Ferritin	Valid N	0	15	19	0	0	14	10	5	8
	Valid Percent	0%	6.7%	0%	0%	10.5%	0%	0%	20%	25%
Folate	Valid N	2	12	12	9	17	15	11	5	7
	Valid Percent	0%	0%	0%	11.1%	0%	0%	0%	0%	0%
Vit B12	Valid N	2	17	18	15	21	15	12	6	7
	Valid Percent	0%	17.6%	5.6%	6.7%	9.5%	6.7%	16.7%	0%	14.3%
Vit D	Valid N	1	10	13	12	16	13	11	5	8
	Valid Percent	100%	80%	76.9%	75%	87.5%	84.6%	100%	100%	100%
Calcium	Valid N	0	20	19	15	20	15	10	0	7
	Valid Percent	0%	5%	10.5%	13.3%	0%	6.7%	0%	16.7%	0%
Phosphate	Valid N	0	15	19	15	19	15	10	6	6
	Valid Percent	0%	6.7%	0%	0%	0%	0%	0%	0%	0%
Vit A	Valid N	0	6	7	8	12	9	7	2	3
	Valid Percent	0%	0%	14.3%	12.5%	16.7%	22.2%	28.6%	0%	0%
Vit B1	Valid N	0	5	9	9	11	9	7	2	3
	Valid Percent	0%	0%	0%	0%	0%	0%	0%	0%	0%
Vit B6	Valid N	0	5	1	1	6	5	3	1	2
	Valid Percent	0%	0%	100%	0%	33.3%	0%	33.3%	0%	50%
Vit E	Valid N	0	2	7	6	7	3	0	2	2
	Valid Percent	0%	0%	0%	0%	0%	0%	0%	0%	50%
INR	Valid N	10	17	8	5	9	8	5	2	4
	Valid Percent	0%	0%	0%	0%	0%	0%	0%	0%	0%
Albumin	Valid N	7	16	12	12	18	15	11	6	7
	Valid Percent	0%	0%	16.7%	16.7%	0%	0%	0%	0%	0%
Total Proteins	Valid N	7	21	18	15	21	16	12	6	7
	Valid Percent	0%	0%	22.2%	40%	33.3%	18.8%	25%	0%	0%

a 1 patient had symptomatic gallstone disease and Dumping syndrome.

INR, international normalized ratio; LD, limb distalization; M, months; Vit, vitamin; RYGB, Roux-en-Y gastric bypass.

## 4 Discussion

Guidelines on how to report results after secondary MBS are lacking. Primary and secondary weight loss unsatisfactory response are inconsistently defined in the literature (3). Hence, thresholds for an escalation of therapy by revisional surgery are at the surgeon's discretion (18). Recently, a panel of experts addressed some of these gaps to enable more clear standardization and comparison of results (7).

From 2011 to 2023, our center performed 3,984 MBS, including 3,205 RYGB (unpublished data) and 22 LD (0.6%). Despite the fact that up to 30% of patients may have a suboptimal clinical response (6), only 21 patients underwent LD at our center, including one case referred from another institution, emphasizing how infrequently this procedure is performed even in high-volume centers. Multiple reasons contribute to the small numbers of LD. Firstly, conservative interventions, such as cognitive behavioral therapy, nutritional, and sometimes pharmacological interventions, may be sufficient to manage patients and restore weight loss. Secondly, anatomical factors that may have contributed to weight regain may require a different surgical approach; thirdly, patients may refuse a second MBS; and last but not least, the multidisciplinary team evaluation may deem patients unsuitable to undergo surgery. The most common reasons why patients were deemed unsuitable often stemmed from the increased risk of nutritional deficits associated with such procedures. These include financial constraints preventing access to an adequate diet for nutritional needs, micronutrient supplements and unwillingness to adhere to the follow-up plan that is required to ensure patient safety.

Although 4 variants of LD have been described (8), there are two main techniques from which the remaining derive. The first, described by Sugerman et al. (19), involves re-anastomosing the jejunojejunostomy to the ileum at 150 cm from the ileocecal valve, leaving a long BPL unmeasured. The second, introduced by Brolin et al. (20), reconnects the BPL at 75 cm from the ileocecal valve, leaving a common channel of 75 cm and a long AL unmeasured. At our institution, the first approach was adopted with a tailored approach to limb length, individualized for each patient. Our technique relies on our previous studies that the BPL is important for weight loss and metabolic improvement (14–16). In our cohort, the initial RYGB's BPL length was variable, thus the goal of LD was to extend it to a minimum length of 200 cm, or 300 cm whenever the previous BPL length was already 200 cm. The wide range of values shown in Table 1 reflects the application of this approach to the total length of the small intestine in each patient (21). The length of the BPL will determine the total alimentary limb length (TALL), with an aim of 300 cm to prevent short bowel manifestations, such as the risk of diarrhea and nutritional deficiencies.

The optimal limb length is widely debated in literature. The line between sufficient weight loss and nutritional deficiencies is extremely narrow.

In our cohort, we achieved a statistically significant reduction in mean weight and BMI in the short-term (≤1 year), medium-term (2–4 years), and long-term (≥5 years), results that are in line with other published studies (19, 22–33). At 12-month follow-up, the mean %EWL and %TWL were 58.2% and 19.6%, respectively, consistent with previously reported values (24, 26, 27, 29, 30, 32, 33). Similarly, at 60 months or more of follow-up, an %EWL of 49.9% and %TWL of 19% are within the range of published values (24, 28, 30, 32, 33). When comparing our results to the %TWL < 20% threshold recommended for defining a suboptimal clinical response to MBS in recent consensus guidelines (7), several factors must be considered. Firstly, patients can achieve metabolic benefits with a %TWL > 10% (34). Secondly, this threshold (%TWL < 20%) applies to primary MBS, whereas expected outcomes for revisional surgery are generally lower. Similarly, if we apply other criteria from the literature for primary surgery, such as %EWL > 50% as proposed by Kim E. (2), the results fall within the range of “therapeutic success”. Thirdly, and most importantly, LD represents a continuum in the surgical treatment of a chronic disease–obesity. When %TWL values are compared to the patient's initial weight (pre-RYGB), the long-term %TWL is 28.3%.

In terms of nutritional deficiencies, the most significant ones were vitamin D deficiency, iron-deficiency anemia, and protein malnutrition. LD does not seem to have a greater deleterious impact on vitamin D levels or iron stores, since 78% of healthy people in the region are reported to be vitamin D deficient (35) and 60.5% of patients after RYGB develop iron-deficiency anemia (6). This highlights the importance of proper patient selection for LD, likely favoring those patients who are more compliant, as well as adequate follow-up for potential adjustments. Regarding protein deficiencies, we observed only 2 patients (9.1%) with serum albumin levels below 3.4 g/dL. One patient was amenable to oral supplementation, while the other required hospitalization for parenteral nutrition at 12 months after surgery. This rate is considerably lower than reported in some studies, where surgical intervention was even necessary to reverse such cases (19, 22, 24–30, 32, 33). These rates appear particularly high in studies where the TALL was shorter (250 cm) and the common limb (CL) measured 100 cm (28, 30, 32). Both of our patients had a TALL of 300 cm and a CL of 100 cm. In fact, there is evidence that a shorter TALL results in greater weight loss but increases the risk of nutritional deficiencies, especially when combined with a short CL (36). Given the close relationship between the BPL and TALL, it is reasonable to consider that the key to enhance weight loss lies in increasing the length of the BPL, which has been shown to be associated with greater weight loss and obesity-related diseases remission after RYGB (37). Conversely, the length of the AL does not seem to significantly affect weight loss (36). We believe that in LD, we should focus on finding the ideal solution for the patient rather than for the limb. Pre-existing anatomy, compliance, and eating habits should be considered in this decision-making process. A TALL of 300 cm seems safe, provided that close and adequate follow-up and micronutrient supplementation are feasible.

Regarding the procedure itself, the median operative time was 76 min with only 2 cases lasting more than 100 min. The median hospitalization duration was 2 days, and the early complication rate was 0%. These values fall within the expected ranges based on benchmark cut-offs (38), though caution is warranted due to the small sample size. Before attributing the 27% (6/22) late complication rate to LD, one must critically interpret it. Indeed, all these complications are well-documented as late complications of the MBS, particularly RYGB. Whether these events are related to LD or would have occurred anyway is unknown.

Analyzing the results at 10 or more years of follow-up after RYGB, previously published by our group, we can conclude that patients with baseline BMI above 45 kg/m^2^ were associated with the worst long-term weight loss results (6). At the time of index surgery, the patients who underwent LD had a mean BMI of 45.9 ± 5.5 kg/m^2^. This finding suggests that the indication for RYGB in patients with BMIs above 45 kg/m^2^ warrant reconsideration, given the greater probability of suboptimal long-term results.

LD was associated with excellent results regarding improvement and/or remission of metabolic diseases in our series. This could be an additional indication for LD technique. The basis for metabolic improvement could be related to the lengthening of BPL in all patients, promoting a more significant postprandial response of GLP-1 and neurotensin (14, 15).

How does LD outcomes compare to other techniques, especially with the conversion of RYGB to SADI-S or DS ± BPD, is unknown as there are no reports in the literature that provide such direct comparisons. However, these techniques seem to entail a considerable number of staged surgeries due to its complexity (39). The %TWL at 24 months seem to be higher with these techniques [Surve et al. 29.2% (40), Moon et al. 25.7% (39)], but there is also an important rate of short-term complications [13.3 (39)–25% (40)], including reoperations. The remission rates of obesity-related diseases and nutritional deficits were comparable to those reported in this study (39).

Finally, pharmacological options have emerged as a viable option for patients with recurrent weight gain after MBS, especially for those who are not candidates for secondary surgery. These medications act by targeting different physiological pathways that influence appetite and metabolism (41). Despite the lack of comparative studies in patients with recurrent weight gain, LD appears to be associated with a greater and more sustained reduction in TWL when compared to what is achieved by GLP-1R agonists (42).

Lifelong post-operative monitoring of patients must be carried out in order to prevent or enable early diagnosis of possible micronutrient deficits. The good results in this cohort of patients seem to be influenced by this successful follow-up with no drop-offs.

Our study has several important limitations, primarily related to its retrospective design, which is inherently subject to selection and reporting biases. Final BPL length was not systematically documented in operative reports throughout the study period, therefore, reliable cohort-level BPL data could not be reported. A major limitation is the lack of systematic documentation of final BPL length in all patients. This is particularly relevant because the rationale of our LD technique is based on increasing the BPL while maintaining an adequate TALL. Although BPL length can be measured intraoperatively at the time of LD, and, in some cases, calculated from total small bowel length, AL length, and CL length, these variables were not consistently recorded in the operative reports during the entire retrospective study period. Consequently, final BPL length could not be reliably determined for the whole cohort. This limits comparison with other LD series and prevents a direct analysis of the association between BPL length and weight loss, metabolic outcomes, or nutritional complications. Future prospective studies should include standardized intraoperative measurement and documentation of BPL, AL, CL, TALL, and total small bowel length. In addition, the highly selective group of patients, likely representing those who were more motivated and compliant, could have influenced the results. However, it is our practice to restrict the access to LD surgery of patients with a higher potential of developing micronutrient deficiencies. Another limitation was the small sample size, in common to previous studies found in literature, which is also a consequence of patient selection. This limitation, along with the single-center design, significantly limits the generalization of the findings. Lastly, at the time of observation only 40.9% of the patients had so far reached long-term follow-up. Therefore, it is advisable to interpret the results with caution having this time in mind.

## 5 Conclusion

In our study, LD was associated with a substantial weight reduction and significant remission of obesity-related diseases, such as T2D, dyslipidemia, and AHT. Despite the limited sample size, there were no complications in the first 90 days after surgery, and the rates of long-term complications were identical to those described for primary MBS. Proper follow-up is a cornerstone in reducing the rate of micronutrient deficiencies. Further research with larger sample sizes is necessary to validate these findings and enhance comprehension of long-term results.

## Data Availability

The raw data supporting the conclusions of this article will be made available by the authors, without undue reservation.
